# Hernias of the Uterine Appendages

**Published:** 1913-01

**Authors:** Aime Paul Heineck

**Affiliations:** Surgeon to the Cook County Hospital, Chicago


### HERNIAS OF THE UTERINE APPENDAGES

By Aime Paul Heineck, M. D.

*Surgeon to the Cook County Hospital, Chicago*

(Concluded from October Number, 1912.)

### Femoral Hernias.

Hernias which in their escape from the abdominal cavity pass between Poupart's ligament and the horizontal ramus of the pubis and sooner or later protrude in Scarpa's triangle, are called femoral hernias. Common femoral hernias escape from the abdomen through an orifice bounded anteriorly by the most internal portion of Poupart's ligament; posteriorly, by the horizontal ramus of the pubis; externally, by the femoral vein and the sheath of the femoral vessels; internally, by Gimbernat's ligament They descend along the most internal compartment of the femoral sheath and ultimately emerge through the saphenous opening. These hernias are contiguous to the femoral vein which always lies external, and they carry along in their progress through the crual canal a mass of condensed areolar tissue, known as the septum crurale. They show a greater tendency to expand upward than downward because the cribriform fascia is less adherent to the upper margin of the saphenous opening.

The small number (sixteen cases) of femoral hernias which we were able to collect, as compared to inguinal hernias, confirms the now accepted but previously disputed fact that, in the female, inguinal hernias are of more frequent occurrence than femoral hernias. All these femoral hernias were of the acquired type; it is known that congental femoral hernias are pathological rarities. A femoral hernia is essentially a hernia of adult life. Either side of the body may be involved. The tendency to double femoral hernia is less than that to double inguinal hernia. All femoral hernias irrespective of contents or of sex of bearer are of more frequent occurrence on the right side (Wernher, Macready, Berger).

The tubal hernias contained the oviduct either in part or in its entirety, alone or associated with intestine, omentum or both. The presence of the appendix vermiformis in a femoral hernial sac is rare. Coley states that in 2200 cases of hernia operated upon from 1890 to March 1908 in the Hospital for Ruptured and Crippled, the appendix vermiformis alone was found in ten, the cecum and appendix together in seven. In not a single one of Coley's cases was the appendix found in a femoral hernial sac (W. B. Coley, Keen's Surgery, 1908, vol. I, p. 78).

These hernias may be reducible, irreducible or strangulated. Strangulation can occur at any one of the following sites:—

(a). Internal femoral ring.

(b). Margin of the Gimbernat's ligament.

(c). Margin of the saphenous opening.

(d). Meshes of the cribriform.

(e). Irregularities in the hernial sac.

*The following operations were performed*:-

(a). Amputation of fimbriated end of tube.

(b). Incision of hernial swelling and creation of an artificial anus.

(c). Ablation of the tube.

(d). Ablation of ovary.

(e). Ablation of tube and ovary.

(f). Excision of hernial sac and return of tube to abdominal cavity.

(g). Removed tube and ovary and resected gangrenous intestine.

Truss treatment of femoral hernia is notoriously unsatisfactory and is considered as being only palliative and not at all curative. In femoral hernias, on account of motions of thigh, it is difficult to apply, and especially to maintain pad-pressure in position conductive to closure of the hernial openings.

Coley whose clinical experience with the treatment and cure of hernia is greater than that of any other living American surgeon, supplements to high ligation of the hernial sac with the thorough removal of all extra-peritoneal fat the following step:- He introduces a purse-string suture of kangaroo tenden in such a way as to bring the floor of the femoral canal in contact with the roof.

### Inguinal Hernias.

Hernias which escape from the abdominal cavity, either through the internal or external inguinal fossae, and which emerge upon the surface when complete by way of the external abdomnal ring, are known as inguinal hernias. Of all hernias, they are the ones most frequently noted in the female.

Inguinal hernias of the uterine adnexae may be completely or incomplete. In the incomplete form, the hernia has not escaped beyond the external abdominal ring. Inguinal hernias may be right-sided or left-sided. They may be unilateral or bilateral; as previously stated, all the bilateral hernias of the uterine adnexas, tubal, ovarian or tubo-ovarian, recorded in the medical literature of the last twenty years, were of the inguinal variety. They may be reducible or irreducible. They may be strangulated or the seat of torsion. This last accident has only been observed in congenital inguinal hernias, the contents of which were irreducible.

Of the uncommon types of inguinal hernias only several could be found. There was one direct hernia. This especially in the female, is an uncommon form. The main characteristics of direct hernias are that the protrusion takes place by way of the internal fossa, that the neck of the sac is always to the inner side of the deep epigastric vessels and, in the female, that the round ligament is distinct from and to the outer side of the sac. We found three interstitial or intra-parietal hernias. All the other inguinal hernias were of the ordinary type, that is, external or indirect or oblique. An ordinary or oblique inguinal hernia in its course through the inguinal canal (narrower in the female than in the male) is accompanied by the round ligaments; as it escapes from the external abdominal ring, it appears in the upper portion of the labium majus, in which it descends to a greater or less extent.

Inguinal hernias vary in size and in form. They may be almond-sized, ovoid, sausage-shaped, pear-shaped, pyriform, globular or other forms too numerous to mention.

Symptoms and Diagnosis.

Hernias of the uterine appendages present all the symptoms common to hernias in general. In some hernias of slow and gradual development, owing to the absence of symptoms, not uncommonly the patient ignores that he has a rupture. Some cases do not give rise to any symptoms; some give rise to very slight disturbances; many remain until the appearance of the menses and thus are first recognized at about the age of puberty. Pain may be absent during the entire course of a slowly developing hernia. In other cases, the hernial swelling may be so small as to be completely overlooked by a careless observer. Inspection, palpation and percussion of the hernial regions are routine steps in the examination. After having demonstrated the presence of a hernia, the operator has to determine the type of hernia present and the nature of its contents. The existence of other malformations is to be ascertained as they may be of such a nature as to partially justify the sacrifice of healthy herniated organs.

Clinicians usually do not experience any difficulty in diagnosing a hernia; at times, they are in doubt as to the type of the hernia present in the case at hand. In fatty individuals, the exact position of the hernial neck is difficult to determine. Obturator hernias are rare, and are found internal to the femoral opening; in femoral hernias, the swelling is found to be almost if not entirely below a line extending from the anterior superior iliac spine to the pubic spine.

In the female, the tumor-mass caused by a complete oblique inguinal hernia, even of a moderate size, will cause swelling into the labium majus.

In some hernias of the uterine appendages, the patients complain of a feeling of weight and discomfort. The symptom "tenderness on pressure" we find frequently reported. Some of these hernias are painless, some are so painful as to interfere with the patient's well-being. Impulse on coughing is not infrequently noted. It is not, however, a constant symptom present; it may be absent. Menstrual disturbances are recorded.

If an oblique inguinal hernia be incomplete, that is, if it does not extend beyond the inguinal canal, it may be mistakenly diagnosed tumor of the round ligament, bube, epiplocele or encysted hydrocele of the canal of Nuck. In tumors of the round ligament, epiploceles, encysted hydroceles of the canal of Nuck, a mistake in diagnoss is not very significant, as in all these conditions one must, to obtain cure, resort to operative treatment

(j). Distortion beyond recognition.

(k). Such pathological changes as prevent function.

SUMMARY.

1. The Fallopian tube, the ovary or the tube and ovary, in part or in their entirety, may be herniated.

2. The herniated tube, ovary or tube and ovary may be the sole content of the hernial sac or there may be present as associated hernial contents one or two or more of the following structures or organs:- Meckel's diverticulum, appendix vermiformis, omentum, urinary bladder, intestine (small or large), uterus.

3. Tubal, ovarian and tube-ovarian hernias are congenital or acquired, unilateral or bilateral; exist alone or in association with one or more other hernias of the same or of dissimilar anatomical types, of the same or of dissimilar clinical characteristics.

4. These hernias, in a small proportion of cases, co-exist with malformations, under-development or absence of other internal genitalia or of some external genitalia:- Imperforate vagina, absence of vagina, atresia of tube, unilateral absence of tube, of ovary or of tube and ovary, absence of cervix uteri, rudimentary uterus, absence of uterus, etc.

5. In individuals having a hernia of a tube, an ovary or of a tube and ovary, pathological states of other internal genitalia or of some external genitalia may be present:- Vaginitis, ovarian cystoma, uterine fibrod, uterine prolapse and other uterine displacements, etc.

6. These hernias may co-exist with pathological states of organs other than the internal or external genitalia:- Chronic hydrocephalus, multiple stenosis of intestines, hydronephrosis, etc. these co-existing pathological states having no relation of cause or effect to the hernial infirmity.

7. Congenital or acquired hernias of the tube, ovary or tube and ovary, may develop at any period of life. These hernias have been observed in nulliparae, in primiparae and in multiparae. No age is exempt. No race is immune.

8. According to their anatomical site, hernias of the uterine appendages are designated as post-operative or ventral, gluteal, sciatic or ischiadic, obturator, femoral and inguinal.

9. The tube, the ovary or the tube and ovary may be present alone or in association with other organs in the sac of any variety of gluteal, obturator, femoral or inguinal hernias.

10. Clinically, these hernias are reducible, irreducible, non-inflamed, inflamed, strangulated or their pedicle may be the seat of torsion.

11. Torsion of the pedicle of a herniated ovary or of a tube and ovary, an accident peculiar to, and not infrequent in, hernias of the uterine appendages, gives the same clinical symptoms and determines the same anatomical changes in the herniated organs as are observed in the strangulation of hernial contents at one or another or more points.

12. We were able to collect eight times as many hernias of the inguinal type as of all the other hernial types put together.

13. Tubal, ovarian and tubo-ovarian inguinal hernias are recent, old or recurrent; are direct, interstitial or intra-parietal, indirect or oblique. If indirect or oblique, they are either complete or incomplete. A few sliding hernias are on record.

14. All the bilateral tubal, ovarian or tube-ovarian hernias recorded in the medical literature of the last twenty years were of the inguinal variety. In bilateral hernias, both hernias may or may not show the same degree of development; they may have appeared simultaneously or one may have appeared a shorter or longer time before the other. They may show similar or dissimilar clinical characteristics. When bilateral, one hernia may be irreducible and the other reducible.

15. All the hernias in which the complication "torsion of the pedicle" occurred were irreducible congenital inguinal hernias.

16. All the femoral tubal, ovarian or tubo-ovarian hernias recorded in the medical literature of the last twenty years were of the acquired type and appeared in advanced adult life. "Femoral hernia is essentially a hernia of adult life".

17. Hernias of the uterine appendages, in the absence of anomalies of the other internal genitalia or of the external genitalia, do not prevent conception, do not interfere with gestation, nor unfavorably influence parturition. Pregnancy can occur previous to, or during and subsequent to the existence of hernias of this nature.

18. The etiology of hernias of the uterine appendages is that of hernia in general. As main factors should be cited:- All conditions that weaken the abdominal wall, all conditions that increase the intra-abdominal pressure and all conditions that increase the mobility of the uterine adnexae. Heredity, pregnancy and the partial or complete persistence of the canal of Nuck are the most important predisposing causes.

19. The herniated organ or organs may be free from all degenerative changes.

20. The herniated organ or organs may be bound to the sac-wall, or to each other; may be the seat of congestion, gangrene, hemorrhage, inflammation, suppuration, tuberculosis (primary or secondary), cystic and neoplastic disease (benign or malignant).

21. The herniated organ may be the seat of gestation.

22. The hernial sac and the herniated adnexa or adnexae may be the seat of an inflammation, suppuration or other in character, which in progressing by continuity or surface has extended upward from the vagina giving us the following anatomical picture:- Vaginitis, endocervicitis, endometritis, salpingitis or pyosalpinx, ovaritis and saccular peritonitis.

23. The hernial sac and the herniated contents may be the seat of an inflammation, suppurative or other in character, which has reached the tube and ovary by way of the parametrial and parasalpingeal connective tissue.

24. Pathological processes originating in the hernial contents may by extension by contiguity of tissue involve the sac and its overlying tissues.

25. The hernial sac and the herniated tube, ovary or tube and ovary can become the seat of pathological processes secondary to disease of the associated hernial contents. Epiploitis, appendicitis, gangrenous gut, etc. Infection spreading by contiguity of surfaces.

26. The herniated tube, ovary or tube and ovary and the associated hernial contents may be free of disease or the uterine adnexae may be normal and pathological changes be present in the associated hernial contents:- Appendicitis, gangrenous gut, and to exposure of the inguinal canal.

### Treatment.

Six of the reported cases were either autopsy-table or dissecting-room discoveries. In some cases, the nature of the operative intervention is not stated. In the other cases, the operators, after performing either a laporotomy or a herniotomy, disposed of the herniated organs either by reducing or removing them entirely or by removing a part and reducing the remainder. In some cases, the reduction of the hernial contents necessitated a preliminary division or a dilatation of the hernial rings or external.

In two hundred and thirteen operated cases the results are stated: Eleven deaths; two hundred and two recoveries.

We advise that all hernias of the uterine appendages, irrespective of anatomical site or of size or of age of bearer, be subjected to an operation for radical cure:—

(a). If the hernia be irreducible.

(b). If the hernia be strangulated.

(c). If the pedicle of the herniated organ or organs be the seat of torsion.

*After the age of two years—*

(a). If the hernia be bilateral.

(b). If other hernias be co-existent.

(c). When hernia cannot be painlessly, completely and permanently kept reduced.

(d). If organs other than the uterine appendages be also present in the same hernial sac.

(e). If the wearing of a hernial truss causes pain or aggravates the symptoms.

(f). If the patient has to be subjected to ether or chloroform anesthesia for the performance of an operation of election, double advantage can be taken of this anesthesia, and an operation for the radical cure of the hernia performed.

(g). If patient is exposed to pregnancy.

Operation in uncomplicated hernias of the uterine adnexae is no more dangerous than the operation for the radical cure of other hernias. It has practically no mortality. Infants bear hernia operations remarkably well. Broca performed four hundred anl fifty operations in children under fifteen years of age with but one death.

The operation which has given the most universal satisfaction in the treatment of inguinal hernias is that devised by Bassini. It has the advantages of safety, simplicity and efficacy.

We advise operators to observe in their operations for inguinal hernia, the following suggestions:-

1. Always wear, and have the assistants wear rubber gloves.

2. All ligatures and irremovable buried sutures should be of absorbable material. The purpose of sutures is to keep divided tisues in opposition until organic union has been effected. After this has been accomplished, sutures if not absorbed or not removed may originate irritation, may act as predisposants to inflammation, to sinus formation. We strongly condemn the use of silk for vessel ligation or for buried sutures.

3. Always divide the aponeurosis of the external oblique muscle to an extent sufficient to give good exposure of the inguinal canal, and of its contents. In the female, the inguinal canal in its normal state and after a hernia operation, in its restored state, should outside of a few arterioles and nerve filaments, contain nothing but the round ligament, a structure much smaller than the spermatic cord. This round ligament comes from the muscular structure of the uterus, it finally becomes lost in the labium majus. In a hernia operation, if not the seat of disease, it should never be sacrificed.

4. Always make a high and careful dissection of the hernial sac from the surrounding tissues and especially from the round ligament to which it is often quite intimately adherent.

5. Always open the sac and determine by direct inspection and palpation the nature and state of the hernial contents.

6. After reduction or ablation of the hernial contents the sac is to be transfixed and ligated as high as possible. Sac is then removed, flush with the peritoneal cavity. So as to prevent the occurrence of peritoneal bulging at the internal ring, we are in the habit of anchoring or fixing the stump of the sac about two centimeters above this point. This is effected by needling each end of the ligament and carrying both ends separately and about one centimeter apart upwards in front of the pro-peritoneal fatty cellular tissue and behind the transversalis muscle for about two centimeters. The needles and ligature are then passed from behind forward through the transversalis, internal oblique and external oblique muscle, in front of which the ends of the ligature are tied.

7. Never sacrifice the round ligament; it is harmful to the statics of the uterus. Never transplant the round ligament; it is unnecessary. The round ligament is left undisturbed at the bottom of the wound, emerging at the lower angle of the latter; the internal oblique muscle is sutured to the shelving portion of Poupart's ligament; the divided margins of the external oblique aponeurosis are sutured and the skin incision closed. No drainage. After operation, no truss should be worn; a truss does not support the scar, it weakens it.

8. In the female, the internal and external abdominal rings can be closed without detriment to the patient. In direct inguinal hernias, ligation of the deep epigastric artery is at times unavoidable.

In hernias of the uterine appendages, the operator must decide as to whether the hernial contents are to be returned to the abdominal cavity or whether they are to be removed.

As to the hernated tube, ovary or tube, when normal, it goes without saying that they should be returned, irrespective of patient's age; if adherent to sac-wall or to some hernial content, the adhesions are to be loosened or divided, and if the organ or organs do not show marked structural impairment, they are to be reduced. These organs, when herniated, should be removed, if they be the seat of:—

(a). Unavoidable or actual gangrene.

(b). Benign neoplastic disease.

(c). Malignant neoplastic disease.

(d). Voluminous cyst-formation.

(e). Malformation or incomplete development.

(f). Suppurative inflammation.

(g). Hematoma or interstitial ovarian hemorrhage.

(h). Seat of tubal gestation previous or subsequent to rupture of fetal sac.

(i). Tuberculosis limited to or extending beyond the herniated organ.

(j). Distortion beyond recognition.

(k). Such pathological changes as prevent function.

### Summary

1. The Fallopian tube, the ovary or the tube and ovary in part or in their etirety, may be herniated.

2. The herinated tube ovary or tube and ovary may be the sole content of the herinal sac or there may be present as associated hernial contents one or two or more of the following structures or organs; Meckel's diverticulum, appendix vermiformis, omentum, urinary bladder, intestine (small or large), uterus.

3. Tubal, ovarian and tube-ovarian hernias are congenital os acquired, unliateral or bilateral; exist alone or in association with one or more hernias of the same or of dissimilar anatomical types, of the same or of dissimilar clinical characteristics.

4. These hernias, in a small proportion ot cases, co-exist with malformation, under-development or absence of other internal genitalia or of some external genitalia: Imperforate vagina, absence of the vagina, atresia of tube, unilateral absence of tube, or ovary or of tube and ovary, absence of cervix uteri, rudimentary uterus, absence of uterus, etc.

5. In individuals having a hernia of a tube, an ovary or of a tube and ovary, pathological states of other internal genitalia or of some external genitalia may be present: Vaginitis, ovarian cystoma, uterine fibrod, uterine prolapse and other uterine displacements, etc.

6. These hernias may co-exist with pathological states of organs other than the internal or external genitalia: Chronic dydrocephalus, multiple stenosis of intestines, hydronephrosis, etc. these co-existing pathological states having no relation of cause or effect to the hernial infirmity.

7. Congenital or acquired hernias of the tube, ovary or tube and ovary, may develop at any period of life. These hernials have been observed in nulliparae, in primiparae and in nulliparae. No age is exempt. No race is immune.

8. According to their anatomical site, hernias of the uterine appendages are disignated as post-operative or ventral, gluteal, sciatic or ischaiadic, abturator, femoral and inguinal.

9. The tube, the ovary or the tube and ovary may be present alone or in association with other organs to the sac of any variety of gluteal, obturator, femoral or inguinal hernias.

10. Clinically, these hernias are reducible, irreducible, non-inflamed, inflamed, strangulated or their pedicle may be the seat of torsion.

11. Torsion of the pedicle of a herniated ovary or of a tube and ovary, an accident peculiar to, and not infrequent in, hernias of the uterine appendanges, gives the same clinical symptoms and determines the same anatomical changes in the herniated organs as are observed in the strangulation of hernial contents at one or another or more points.

12. We were able to collect eight times as many hernias of the inguinal type as of all the other hernial types put together.

13. Tubal, ovarian and tubo-ovarian inguinal hernias are recent, old or recurrent; are direct, interstitial or intra-parietal, indirect or oblique. If indrect or oblique, they are either complete or incomplete. A few sliding hernias are on record.

14. All the bilateral tubal, ovarian or tube-ovrian hernias recorded in the medical literature of the last twenty years were of the inguinal variety. In bilateral hernias both hernias may or may not show the same degree of development; they may have appeared simultaneously or one may have appeared a shorter or longer time before the other. They may show similar or dissimilar clinical characteristics. When bilateral one hernia may be irreducible and the other reducible.

15. All the hernias in which the complication "torsion of the pedicle" occurred were irreducible congenital inguinal hernias.

16. All the femoral tubal, ovarian or tubo-ovarian hernias recorded in the medical literature of the last twenty years were of the acquired type and appeared in advanced adult life. "Femoral hernia is essentially a hernia of adult life."

17. Hernias of the uterine appendages, in the absence of anomalies of the other internal genitalia or of the external genitalia, do not prevent conception, do not interfere with gestation, nor unfavorably influence parturiation. Pregnancy can occur previous to, or during and subsequent to the existence of hernias of this nature.

18. The etiology of hernias of the uterine appendages is that of hernia in general. As main factors should be cited: All conditions that weaken the abdominal wall, all conditions that increase the intra-abdominal pressure and all conditions that increase the mobility of the uterine adenexae. Heredity, pregnancy and the partial or compete persistence of the canal of Nuck are the most important predisposing causes.

19. The herniated organ or organs may be free from all degenerative changes.

20. The herniated organ or organs may be bound to the sac-wall, or to each other; may be the seat of congestion, gangeren, hemorrhage, inflammation, suppuration, tuberculosis (primary or secondary), cystic and neoplastic disease (benign or malignant).

21. The herinated organ may be the seat of gestation.

22. The hernial sac and the herniated adnexa or adnexae may be the seat of an inflammation, suppuration or other in character, which in progressing by continuity or surface has extended upward from the vagina giving us the following anatomical picture: Vaginitis, endocervicitis, endometritis, salpingitis or pyosalpinx, ovaritis and saccular peritonitis.

23. The hernial sac and the herinated contents may be the seat of an inflammation, suppurative or other in character, which has reached the tube and ovary by way of the parametrial and parasalpingeal connective tissue.

24. Pathological processes originating in the hernial contents may by extension by continguity of tissue involve the sac and its overlying tissues.

25. The hernial sac and the herniated tube, ovary or tube and ovary can become the seat of pathological processes secondary to disease of the associated hernial contents. Epiploitis appendicitis, gangernous gut, etc. Infection spreading by contiguity of surfaces.

26. The herniated tube, ovary or tube and ovary and the associated hernial contents may be free of disease or the uterine adnexae may be normal and pathological changes be present in the associated hernial contents: Appendicitis ganggronous gout epipleitis, etc. The associated hernial contents may be normal and the herniated uterine adnexae be the seat of morbid changes.

27. It is at times difficult, at times impossible, to state with absolute precision whether the anatomical changes present in the herniated organ or organs developed previous to or subsequent to the displacement of the tube, ovary or tube and ovary into the hernial sac.

28. Truss-treatment for hernias of the uterine appendages is curative, is often productive of discomfort and interferes with the nutrition and development of the herniated tube or ovary.

29. Women who suffer from any form of hernia should be carefully watched before, during and after their confinement so as to prevent or rather minimize any undue strain upon weak regions of the abdominal wall. These women, at the close of lactation or towards the end of the first year following their confinement, should in the absence of contra-indications be subjected to an operation for radical cure of the hernia.

30. In the female, all hernias irrespective of anatomical site, of clinical condition or nature of contents, should, in the absence of a constitutional state contra-indicating operations of election, be subjected to an operation for radical cure.

31. Clinical conditions so closely simulating hernias of the uterine appendages that a postive diagnosis without operation appears impossible, should be subjected to operative treatment. Only benefit can be derived from adherence to this rule. A diagnosis is established and a cure is effected.

32. In these hernias as well as in other hernias. the ideal time for operation is previous to the development of the degenerative or other pathological conditions in the herniated organ or organs and previous to the occurrence of any of the various complications incident to hernias.

33. The mortality of operations for the radical cure of hernias, if performed at an opportune time and by a rapid operator competently assisted, is nil.

34. To be effective, operations for radical cure of hernias must well fulfill two essentials:- The suppression of the sac and the strengthening of the area through which the hernia has escaped.

35. In all herniotomies, the sac should be incised and the hernial contents examined.

36. In the female, the inguinal rings are comparatively small. They can, without inconvenience to the patient, be closed.

37. The herniated normal tube or ovary should never be sacrificed. These organs have an important role and in the absence of marked structural impairment should be returned to the abdominal cavity.

38. The herniated abnormal tube, abnormal ovary or abnormal tube and ovary should be removed if their return to the abdominal cavity is associated with peril, immediate or remote, to the patient or if these organs are so altered anatomically as to be functionally worthless. In sacrificing tissues or organs, the surgeon must be economical.

39. Until we are better informed as to the frequency and nature of true and false hermaphroditism, removed herniated uterine adnexae not having a distinctive structure should be subjected to a microscopical examination. This will avoid mistaking testicular for ovarian tissue and vice versa.

40. In the treatment of strangulated sciatic or gluteal, obturator and femoral hernias of the uterine appendages in which the hernial sac also contains gangrenous gut, a double operation is always indicated: A laparotomy for the repair of the intestinal lesions and a herniotomy for the radical cure of the hernia.

